# A persistently low level of atmospheric oxygen in Earth’s middle age

**DOI:** 10.1038/s41467-020-20484-7

**Published:** 2021-01-13

**Authors:** Xiao-Ming Liu, Linda C. Kah, Andrew H. Knoll, Huan Cui, Chao Wang, Andrey Bekker, Robert M. Hazen

**Affiliations:** 1 Department of Geological Sciences, University of North Carolina, Chapel Hill, NC 27599 USA; 2 Department of Earth and Planetary Sciences, University of Tennessee, Knoxville, TN 37996 USA; 3 Department of Organismic and Evolutionary Biology, Harvard University, Cambridge, MA 02138 USA; 4 Department of Earth Sciences, University of Toronto, Toronto, ON M5S 3B1 Canada; 5 Geomicrobiology Group, Institut de Physique du Globe de Paris, University of Paris, 75005 Paris, France; 6 Department of Earth and Planetary Sciences, University of California, Riverside, CA 92521 USA; 7 Department of Geology, University of Johannesburg, Auckland Park, Johannesburg, 2006 South Africa; 8 Earth and Planets Laboratory, Carnegie Institution for Science, Washington, DC 20015 USA

**Keywords:** Biogeochemistry, Element cycles

## Abstract

Resolving how Earth surface redox conditions evolved through the Proterozoic Eon is fundamental to understanding how biogeochemical cycles have changed through time. The redox sensitivity of cerium relative to other rare earth elements and its uptake in carbonate minerals make the Ce anomaly (Ce/Ce*) a particularly useful proxy for capturing redox conditions in the local marine environment. Here, we report Ce/Ce* data in marine carbonate rocks through 3.5 billion years of Earth’s history, focusing in particular on the mid-Proterozoic Eon (i.e., 1.8 – 0.8 Ga). To better understand the role of atmospheric oxygenation, we use Ce/Ce* data to estimate the partial pressure of atmospheric oxygen (pO_2_) through this time. Our thermodynamics-based modeling supports a major rise in atmospheric oxygen level in the aftermath of the Great Oxidation Event (~ 2.4 Ga), followed by invariant pO_2_ of about 1% of present atmospheric level through most of the Proterozoic Eon (2.4 to 0.65 Ga).

## Abstract (web summary)

Constraining the rise in atmospheric oxygen through the early Earth is important to understand the evolution of complex life. Here, the authors find that a major rise in atmospheric oxygen level occurred after the Great Oxidation Event, followed by pO_2_ within 1% of present atmospheric level through most of the Proterozoic Eon (2.4 to 0.65 Ga).

## Introduction

Earth’s O_2_-rich atmosphere, unique among known planets, has played an essential role in the evolving feedbacks between life and the environment. The Proterozoic Eon, nearly 2 billion years in duration, has long been characterized in terms of two major episodes of oxygenation, the Great Oxidation Event (GOE) near the beginning of the eon and the Neoproterozoic Oxidation Event (NOE) near its end^1,2^. Between these two events, however, the level of oxygen in the atmosphere and surface ocean, as well as the vertical distribution of oxygen in the oceans, remains a topic of debate. Recently, several attempts have been made to constrain the atmospheric oxygen levels during the mid-Proterozoic. For example, biomarkers from the ca. 1.4 Ga Xiamaling Formation, China, have been interpreted in terms of partial pressure of atmospheric O_2_ > 4 to 8% PAL^3–5^. These values are consistent with previous estimates of the mid-Proterozoic atmospheric. O_2_ levels based on paleosol records^1,6^; however, and other geochemical tracers (Cr isotopes and Ce anomalies) in marine sedimentary records seem to suggest lower pO_2_ (<0.1–1% PAL)^7–9^. Additional geochemical proxy data from marine carbonate phases suggest atmospheric O_2_ levels intermediate between these estimates^10,11^. Most of these studies were based on individual stratigraphic units and did not provide long term estimates of pO_2_ through time. Thus, there remains considerable uncertainty in atmospheric O_2_ level, the degree to which it may have varied through the Proterozoic, and its effects on marine oxygenation and life. Constraining atmospheric oxygen concentration is thus a critical step in understanding the extent to which ocean redox through time was coupled with, or decoupled from, atmospheric evolution^12,13^. Consequently, new quantitative approaches are needed to construct a continuous record of O_2_ level in the mid-Proterozoic atmosphere.

Because marine carbonate rocks incorporate rare earth elements plus yttrium (REE+Y, or REY), REYs have long been used as a paleoredox tracer for ancient shallow-marine environments^14,15^. The cerium anomaly, designated as Ce/Ce* (where Ce/Ce* = Ce × Nd/Pr^2^;^16^), is used routinely to delineate the differential redox behavior of Ce relative to its neighboring REEs and thus fingerprints oxic versus anoxic depositional conditions^14,15^. Under oxic conditions, Ce is preferentially removed from the water column through scavenging by manganese oxides and hydroxides^17^, resulting in a negative anomaly (Ce/Ce* < 1). Under suboxic or anoxic conditions of the modern ocean, Ce anomalies are weak to absent, reflecting the reductive dissolution of Mn-rich and Fe-rich particles below the redoxcline^18,19^; therefore, Ce/Ce* shifts closer to 1. For this reason, Ce anomalies are useful for distinguishing oxic depositional environments from suboxic and anoxic settings^20^.

Recently, REYs in marine carbonates have been used as a proxy for past atmospheric oxygen levels^7,21^ by assuming that the oxygen content of well-mixed surface seawater is in equilibrium with the overlying atmosphere. Wallace et al.^21^ focused on Ce/Ce* in a compilation of marine carbonate rocks deposited between 760 Ma and the present. Bellefroid et al.^7^, in turn, compiled Ce/Ce* data from 1.6 to 0.5 Ga, including data from Wallace et al.^21^, and generated new measurements for ~1.9 Ga platform carbonates from Great Slave Lake, Canada. The modeling performed by Bellefroid et al.^7^ was based on Ce oxidation kinetics, combining water-mass residence time with Ce oxidation and particle adsorption rates. Limited understanding of Ce oxidation kinetics, however, required Bellefroid et al.^7^ to make several assumptions, including critical parameters such as dust flux, particle sinking rate, the redox structure of the mid-Proterozoic ocean, and Ce residence time at different depositional depths. More importantly, they used a global circulation model for the modern ocean to calculate key parameters in the Proterozoic ocean. By contrast, thermodynamics-based Ce oxidation has been widely studied (Supplementary Discussion)^22^, and provides a complementary approach to kinetics-based modeling that requires a number of subjective assumptions. Indeed, a thermodynamics-based approach using Ce data for modern settings generates atmospheric oxygen estimates that are close to the modern atmospheric oxygen level^23^.

This work uses the thermodynamic behavior of Ce/Ce* incorporated into shallow-marine carbonate rocks to quantify ancient atmospheric oxygen levels, focusing mainly on mid-Proterozoic samples (i.e., 1.8–0.8 Ga; broadly between the GOE and the NOE), for which previous estimates of atmospheric oxygen vary markedly. In modeling new and existing data, we make four principal assumptions: 1) shallow-marine environments are well-mixed and in equilibrium with the atmosphere in terms of dissolved oxygen; 2) Ce^3+^, Pr^3+^, and Nd^3+^ have similar solubilities in the oceans; 3) the relative partitioning of Ce vs. Pr and Nd into carbonate minerals has remained the same through time; and 4) oxidation of Ce^3+^ to Ce^4+^, inhibits incorporation into carbonate, whereas Pr and Nd are trivalent and their incorporation behavior is redox-independent.

## Results

### Sample selection and data filtering

In all carbonate samples, the potential for diagenetic alteration is of concern. Fortunately, previous studies provide strong evidence that 1) non-skeletal carbonates can faithfully record the REE composition of ambient seawater^15,24^ and 2) REEs in carbonate rocks are relatively immobile during fluid-rock interaction and can retain a seawater signature even after dolomitization^24,25^, the most widespread diagenetic process to affect carbonate rocks. To limit the potential for carbonate alteration, we selected units with known sedimentological and stratigraphic context, and further investigated the potential for diagenesis via petrographic analysis and elemental and isotope geochemistry. Samples used in this study primarily consist of finely crystalline limestone and penecontemporaneous, fabric-retentive dolostone associated with petrographically well-preserved marine precipitates (e.g., micrite, microbialites, and marine cement). All sample powders were micro-drilled from polished thick sections to avoid clearly altered phases, secondary vein mineralization, and areas with visible non-carbonate phases.

**Fig. 1:**
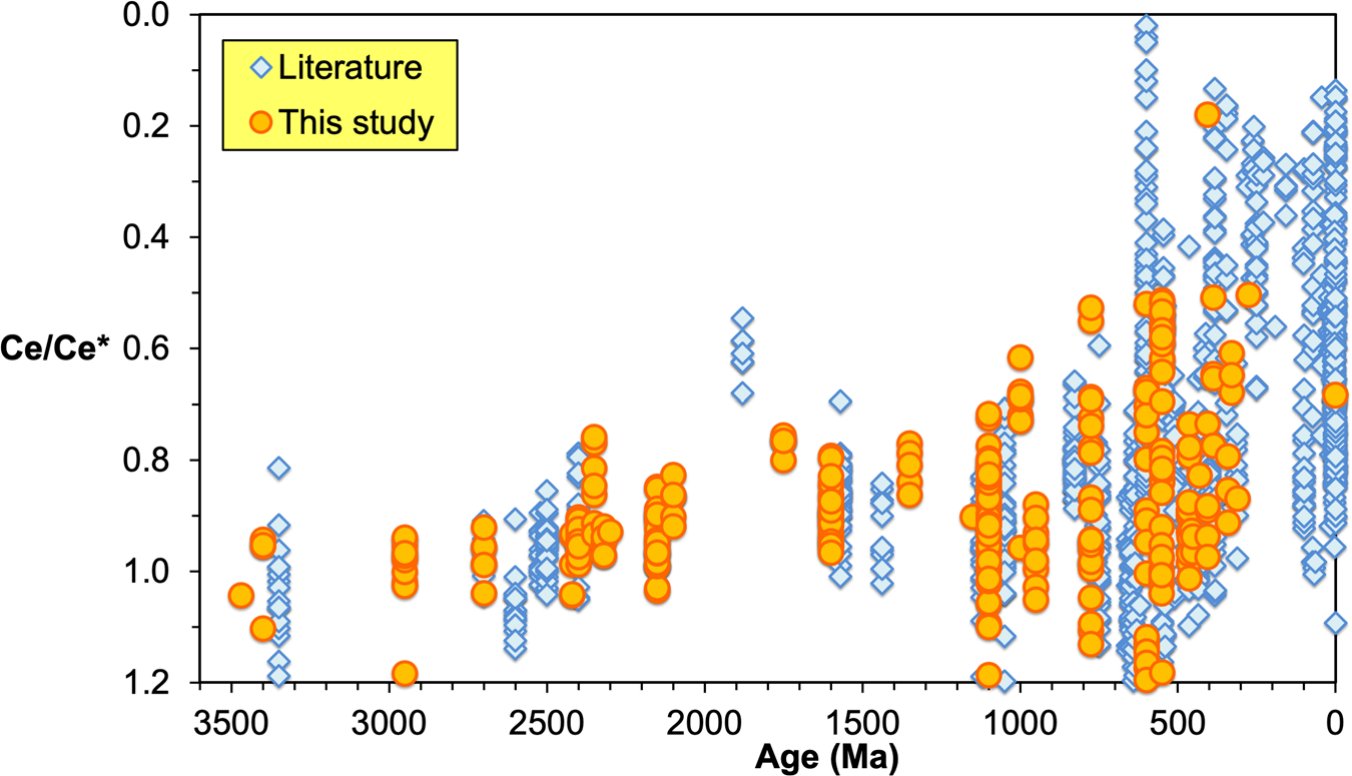
Secular trend of Ce anomaly (Ce/Ce*) in marine carbonates. The figure contains >1300 data points for Ce anomaly, including literature data (blue diamonds) and our 305 new analyses (orange circles).

Even with a carefully selected sample set, the high reactivity of sedimentary carbonate minerals indicates that these samples could have been affected by both syndepositional diagenesis at or near the sediment–water interface and water–rock interactions during burial. Such diagenetic influences can contribute to the local variation of Ce/Ce* (Fig. 1). We acknowledge that all carbonates are therefore likely to have undergone at least some degree of diagenesis, which generally decreases the negative Ce anomaly (increasing Ce/Ce* values)^24^. In addition, modern oxidative weathering could result in large positive or negative variations in the Ce anomaly on a small spatial scale^26^. In consequence, we expect that these post-depositional alteration processes would increase the variance of Ce/Ce* within individual time intervals. Additionally, because REY has a short residence time in seawater, typically less than 1000 years^27^, variation in observed Ce/Ce* can also reflect basin restriction and a decrease in mixing with open-marine surface water. Moreover, Ce anomalies show vertical variability in modern oceans, reflecting dissolved oxygen concentration (see e.g.,^28^). Thus, differences in water depth recorded by shallow-marine carbonates can also contribute to observed Ce/Ce* variation in the dataset, although essentially all of our samples are inferred to have been deposited beneath well-mixed surface water masses. Therefore, selecting samples from relatively shallow-marine settings reduced risk of significant depth influence on the measured Ce anomalies.

In addition to diagenesis, basin restriction, depositional depth, and sample preparation and dissolution can impact the REE signal because carbonate rocks can contain non-carbonate phases such as clay, iron, and manganese (oxyhydr)oxides. Non-carbonate phases could significantly influence the REY concentrations and reduce the magnitude of Ce anomalies, requiring dissolution methods that attack predominantly authigenic carbonate^29,30^. Besides the effects of diagenesis and depositional depth, we consider that non-carbonate contamination has an important impact on Ce anomaly in ancient carbonates. Non-carbonate contamination generally decreases deviation from the non-fractionated Ce/Ce* value of 1^24^; this effect is difficult to exclude from the literature data that we compiled due to a large range of analytical methods and leaching protocols involved. We thus infer that the trend in the lowest Ce/Ce* data, rather than mean values, is most likely to record a true secular change.

We also consider the potential influence of carbonate mineralogy on Ce anomaly data. To address that, we plotted all carbonate samples based on their mineralogy in Supplementary Fig. 3; these data show that there is no systematic difference in Ce/Ce* values between limestone and dolostone through time. This similarity likely reflects the observation that Proterozoic dolostone typically formed at or near the sediment–water interface, preserving depositional or near-depositional geochemical signatures (e.g., see ref. ^31^); neomorphosed limestones (i.e., those that underwent penecontemporaneous recrystallization within the depositional environment) also appear to preserve seawater REE signatures^32^.

In addition to reported geological, petrographic, and geochemical criteria, we further filtered samples based on individual REY patterns and Y/Ho ratios (e.g., see ref. ^19,30^, see also Supplementary Discussion), which could indicate potential post-depositional alteration and non-carbonate phase contamination where such data were available. In this study, we have analyzed more than 400 ancient carbonate rocks, but only 305 samples passed our combined REY and Y/Ho filter criteria (Supplementary Discussion). The only literature exceptions are data from Wallace et al.^21^, where we directly used their reported Ce anomaly data without further filtering. After filtering, we report Ce/Ce* from more than 1300 samples, focusing primarily on Proterozoic carbonate samples (see Supplementary Dataset 1). Limited data available for some published studies, specifically where Y or Ho concentrations were not reported, did not permit data filtering.

## Discussion

**Fig. 2:**
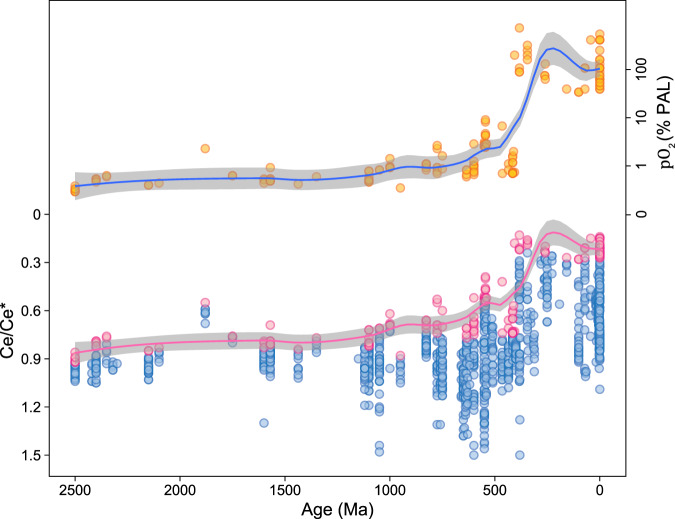
Estimated partial pressure of atmospheric oxygen and Ce anomaly through Earth’s history. Lower part: Secular trend of Ce anomaly (Ce/Ce*) for all complied carbonate data with fitting to lowest 10% of the data shown in pink circles. The pink line indicates the best fit for 100 Ma intervals using the Local Polynomial Regression (LOESS) curve fitting method and the gray field indicates the upper and lower bounds based on 95% confidence level. Upper part: Estimated partial pressure of atmospheric oxygen, pO_2_ (% PAL), through Earth’s history. The blue line shows our best estimate, and the gray field indicates the upper and lower bounds based on 95% confidence level using the LOESS curve fitting method. The pO_2_ was calculated using the lowest 10% of all carbonate Ce/Ce* data for 100 Ma intervals.

Our compilation of Ce/Ce* in marine carbonate rocks is displayed in Fig. 2, with a curve fit to the lowest 10% of Ce/Ce* values in every 100 Myr interval. Similar to previous compilations, our data show a trend of increasing magnitude of negative Ce anomalies towards the GOE and a broad, though modest, increase in the latter part of the mid-Proterozoic interval that accelerated into a marked Ediacaran to mid-Paleozoic change. The slight increase in the negative Ce anomaly after the GOE likely reflects an increased atmospheric oxygen level. In turn, the invarient Ce anomaly in the late Paleoproterozoic to mid-Proterozoic interval suggests that global redox conditions did not increase significantly or irreversibly between the GOE and NOE. The abrupt excursion in the late Neoproterozoic agrees with the timing of the NOE, while the dramatic increase in the lower to middle Paleozoic (~400 Ma^25^) is consistent with independent inferences of whole-scale ocean oxygenation at this time^33,34^.

We use Ce/Ce* data to quantify atmospheric oxygen levels after the GOE because Ce/Ce* is sensitive to distinguishing oxic from suboxic and anoxic environments^20^. Using our compiled Ce/Ce* carbonate data and a thermodynamics-based Ce oxidization model, we provide quantitative estimates of Earth’s atmospheric oxygen evolution after the GOE. Details of the equations and their derivations are provided in Supplementary Discussion. Using the presence of marine carbonate since at least ~2.9 Ga as evidence for buffered marine pH, we infer that pH of the ocean has not changed significantly since the Proterozoic Eon. Nonetheless, small pH change could influence pO_2_ estimate. Thus, we include a sensitivity test of pH in Supplementary Discussion to demonstrate that previously suggested pH range does not significantly impact our conclusions. The influence of temperature on the equilibrium constant (K) in low-temperature surface processes is minor. Therefore, we assume that neither pH nor K changed significantly since the beginning of the Proterozoic in ways, that would significantly affect our conclusions. We can then simplify the relationship between Ce/Ce* and pO_2_ by assuming that the relationship between modern atmospheric pO_2_ and modern seawater Ce/Ce* is reflected in the Ce/Ce* values of the present-day shallow-marine carbonates. Assuming a similar relationship between past carbonate Ce/Ce* values and atmospheric pO_2_, we can calculate pO_2_ for any given time in Earth’s history from Ce/Ce* values preserved in shallow-marine carbonate deposited at that time.

Because secondary processes such as diagenesis and silicate phase contamination drive Ce/Ce* towards unity, we focus on the lowermost values. In addition, the variation recorded within a single time period is expected to reflect the potential redox diversity in depositional environments. For example, Ce/Ce* in modern oceans reflects much higher dissolved oxygen in shallow-water masses than in waters below the redoxcline^28^. Thus, we selected lowest Ce/Ce* values for a given time interval and considered the statistical distribution of sample numbers. We then used the relationship between Ce/Ce* in carbonates and pO_2_ to estimate atmospheric oxygen levels in the past.

We adopted two approaches to estimate the atmospheric oxygen levels: 1) A discrete method that divides data into four groups (Supplementary Discussion) and 2) a smoothing method using 100 Myr time intervals. The results of the first method are given in Table 1, where we use the 10th percentiles of Ce/Ce* data for the four individual age groups (2500–1600, 1600–650, 650–400, and 400–0 Ma) to calculate most probable pO_2_ for these intervals. Groups were defined to reflect the current understanding of pO_2_ evolution through time (see Supplementary Discussion for details), and we additionally explored discrete intervals in the early Phanerozoic (Cambrian-Silurian) and late Phanerozoic (Devonian and later) in an attempt to test existing hypotheses concerning the increase in atmospheric oxygen that resulted from the mid-Paleozoic rise of land plants^21^. Moreover, each group contains samples from at least two different formations to minimize sampling bias. This method suggests that pO_2_ hovered around 1.6% PAL for most of the Proterozoic Eon. Calculations using the 5th and 15th percentiles of Ce anomaly data provide lower and upper bounds of 1.0% and 2.4% PAL, respectively, for pO_2_ (Table 1). We note that the difference between the modern and the past Ce anomaly values determines the magnitude of the oxygen level estimate. Thus, the pO_2_ estimate given by 5th percentiles of Ce anomaly data is lower than that calculated from 15th percentiles of data.

**Table 1:** Atmospheric oxygen level estimates based on the discrete method.

Group	Age	5 percentiles	10 percentiles	15 percentiles
		pO_2_ (% PAL)	pO_2_ (% PAL)	pO_2_ (% PAL)
Paleoproterozoic	2500–1600 Ma	1.0	1.6	2.4
Mesoproterozoic—early Neoproterozoic	1600–650 Ma	0.8	1.6	2.4
late Neoproterozoic—early Paleozoic	650–400 Ma	2.4	5.2	5.5
middle Paleozoic to modern	400–0 Ma	100	100	100

We use 10th percentiles to represent the best estimates, and 5th and 15th percentiles to estimate the lower and upper bounds.

**Fig. 3:**
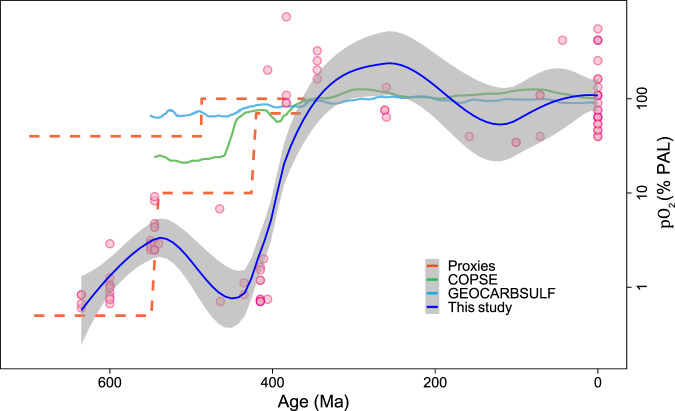
The estimated partial pressure of atmospheric oxygen level *pO*_*2*_ (% PAL) during the Phanerozoic Eon compared to the results of other studies^12,33^. The blue line shows our best estimate, and the gray field indicates the upper and lower bounds based on 95% confidence level using the LOESS curve fitting method. The pO_2_ was calculated using the lowest 10% of all carbonate Ce/Ce* data for 100 Ma intervals during the Phanerozoic Eon.

For the second method, we used the lowest 10% (<10th percentiles) of values for each 100 Myr interval to estimate the atmospheric oxygen level from the Ce/Ce* data for each group; uncertainties are given at the 95% confidence level. This method improves time resolution, but results in greater uncertainties. The pO_2_ curve in Fig. 2 shows the atmospheric oxygen trend inferred from the Ce/Ce* data. We have compared our modeling results during the Phanerozoic Eon with other models and proxy results (Fig. 3). The pO_2_ curve developed in this study is consistent with previous proxy and modeling results, especially for the last ~400 million years^12^. For the earlier Paleozoic Era (541–400 Ma), our atmospheric oxygen level estimate is lower than other estimates^12,33^, perhaps reflecting biases associated with limited sample numbers for this time interval.

Both of these methods provide an estimate of ~1 to 2% PAL for atmospheric oxygen level from the direct aftermath of the GOE to the NOE (Fig. 2 and Table 1). We do not use Ce/Ce* to quantify pO_2_ prior to the GOE because of its low sensitivity under anoxic conditions. Although temperature affects the solubility of O_2_ in seawater, we do not consider variation in temperature in Earth’s history as there is little consensus on the magnitude of temperature change. Lower pH, however, could potentially increase pO_2_ estimates, especially for the mid-Proterozoic, when higher pO_2_ could have resulted in lower seawater pH^35,36^ (see Supplementary Discussion). This potential pH effect requires that our estimates of mid-Proterozoic pO_2_ should be considered conservative and may underestimate atmospheric oxygen levels.

Our calculated values of pO_2_ through time after the GOE are broadly consistent with earlier estimates, although arrived at by different means (Fig. 2). Like other studies, we see little evidence for Proterozoic oxygen increase beyond that achieved during the GOE^37^ until at least the Ediacaran^2^, where our calculations suggest that atmospheric oxygen rose, and remained above 1% PAL (Fig. 2). We do not see evidence for a significant transient atmospheric oxygen increase proposed for the GOE (e.g., see ref. ^2^). However, this result could be due to our low sample density for the GOE. Our calculations suggest an Ediacaran-Cambrian increase in pO_2_ to 2–6% PAL (Figs. 2 and 3)—near the lower end of atmospheric oxygen levels permitted by the interpreted physiology of Lower Paleozoic animals^38^. There also appears to be a transient decrease in atmospheric oxygen level during the Early Paleozoic, which is a time of widespread ocean subsurface anoxia, a limited abundance of skeletal animals, and repeated extinctions^31,39^, before reaching the near-modern atmospheric oxygen levels in the mid-Paleozoic, consistent with oxygenation linked to land-plant evolution.

While we provide atmospheric pO_2_ estimates comparable to previous studies^11,12,38^, we acknowledge some limitations of this thermodynamics-based modeling approach. First, we assume a thermodynamic equilibrium for Ce oxidation, while recognizing that Ce^3+^ oxidization might be kinetically limited. We note that for Ce anomaly, it is not absolute amount that was oxidized is important, but ratio of oxidized to total dissolved Ce. Oxidation rate should scale with pO_2_ and it is a fraction of Ce oxidized rather than absolute amount of Ce that would determine Ce anomaly. Another important assumption that we made is that oxygen content of surface seawater was in equilibrium with the overlying atmosphere. Olson et al.^40^ and Reinhard et al.^41^ proposed that oxygen oases could persist in shallow-marine environments beneath an atmosphere with low pO_2_ as in the mid-Proterozoic. In principle, this could mean that our estimates of early atmospheric oxygen levels are too high. We note, however, that oxygen oases in mid-Proterozoic shallow-marine environments where *P* availability was low and deep waters were ferruginous^41–45^ would likely have been exceedingly limited.

Our best estimate for long-term mid-Proterozoic oxygen levels of ~1 to 2% PAL falls within the lower end of previous estimates^46^. The model of Zhang et al.^3^ infers atmospheric oxygen level between 4 and 8% PAL. Our estimate is higher than those based on Cr isotopes^8,9^, a kinetics-based estimate using Ce/Ce*^7^, and Zn/Fe carbonate-based estimate of Liu et al.^11^. Our thermodynamics-based Ce/Ce* estimate thus supports the general view that atmospheric pO_2_ was one to two orders of magnitude lower than modern content in the mid-Proterozoic^2,6^.

Here we present a novel approach to using the Ce anomaly (Ce/Ce*) measured in shallow-marine carbonates as a proxy for the redox evolution of Earth’s near-surface environments. Because many marine carbonates were deposited in shallow-marine environments, in close contact with the atmosphere, their elemental ratios such as Ce anomaly, as well as zinc to iron ratios^11^, are more likely to reflect the atmospheric redox state. Carbonates provide a continuous record back to the Proterozoic Eon, while other rock records are patchy. More importantly, research on carbonate Ce/Ce* provides a proxy to quantify atmospheric O_2_ throughout Earth’s middle age. Although further work is needed to fully understand this promising paleo-redox proxy, carbonate-based redox proxies show great potential to expand our understanding of redox history and specifically to provide quantitative and self-consistent constraints on atmospheric oxygen levels for the early Earth.

## Methods

### Analytical methods

Rare earth elements plus yttrium (REY) concentrations were measured using a Thermo Scientific^®^ iCAP-Q ICP-MS (Inductively Coupled Plasma-Mass Spectrometry) at the Carnegie Institution for Science. Approximately 5–10 mg of micro-drilled sample powders were weighed and dissolved in 2 ml of distilled 0.4 M HNO_3_ over 12 h. The resulting solutions were centrifuged for 5 min at ~5000×*g*. Then 1 ml of the supernatant from each resultant solution was pipetted and diluted with distilled 4 ml 0.4 M HNO_3_ for elemental analysis. Calibration curves were created using multielemental standards with different dilutions made from pure element solutions (Alfa Aesar®). Both standard and sample solutions were doped with 4 ppb indium to correct for instrumental drift. The precision of the analyses was determined by repeated analyses of an in-house carbonate standard and was typically better than 10% (2*σ*) for rare earth elements plus yttrium (REYs).

## Supplementary information

Supplementary Information

## Data Availability

The authors declare that all source data supporting the findings of this study are available within the paper and its supplementary files. All relevant data are available from the authors upon request. Source data are provided with this paper.

## Source data

Source Data

## Code availability

The authors declare that the code used to analyze and visualize the dataset can be accessed online from figshare (10.6084/m9.figshare.13114379). The Figs. 2 and 3 in the paper were made using R statistical software (http://www.R-project.org/).
