# Supplementary material for: A persistently low level of atmospheric oxygen in Earth’s middle age
Source: Nat Commun. 2021 Jan 13;12:351. doi: 10.1038/s41467-020-20484-7 (PMC7806885; doi:10.1038/s41467-020-20484-7)
Supplement: Supplementary file 1 — Supplementary Information [file 41467_2020_20484_MOESM1_ESM.pdf]

Supplementary Materials for

A persistently low level of atmospheric oxygen in Earth's middle age

Xiao-Ming Liu[1,*], Linda C. Kah[2], Andrew H. Knoll[3], Huan Cui[4,5], Chao Wang[1], Andrey Bekker[6,7], and Robert M. Hazen[8]

[1]*Department of Geological Sciences, University of North Carolina, Chapel Hill, North Carolina 27599, USA*

[2]*Department of Earth and Planetary Sciences, University of Tennessee, Knoxville, Tennessee 37996, USA*

[3]*Department of Organismic and Evolutionary Biology, Harvard University, Cambridge, Massachusetts 02138, USA*

[4]*Department of Earth Sciences, University of Toronto, Toronto, Ontario, M5S 3B1, Canada*

[5]*Geomicrobiology Group, Institut de Physique du Globe de Paris, University of Paris, 75005 Paris, France*

[6]*Department of Earth and Planetary Sciences, University of California, Riverside, CA 92521, USA*

[7]*Department of Geology, University of Johannesburg, Auckland Park 2006, South Africa*

[8]*Earth and Planets Laboratory, Carnegie Institute for Science, Washington DC 20015, USA*

* Corresponding author:

Department of Geological Science

University of North Carolina, Chapel Hill

Chapel Hill, NC 27599-3315

Tel:(919) 962-0675

Email: xiaomliu@unc.edu

Supplementary Discussion

Deviation of equations and sensitivity test

REY complexation in seawater is generally dominated by the formation of carbonate complexes $REYCO_3^+$, and then $REY(CO_3)_2^-$ *(1)*. Therefore, the oxidation of trivalent Ce can be written as below, and similar equations have been derived by Cao et al. (*2*) and references therein:

$$CeCO_3^+ + \frac{3}{2}H_2O + \frac{1}{4}O_2 = HCO_3^- + 2H^+ + CeO_2 \text{ [1]}$$

$$CeCO_3^+ + \frac{3}{2}H_2O + \frac{1}{4}O_2 = CO_3^{2-} + 3H^+ + CeO_2 \text{ [2]}$$

$$Ce(CO_3)_2^- + \frac{3}{2}H_2O + \frac{1}{4}O_2 = 2HCO_3^- + H^+ + CeO_2 \text{ [3]}$$

$$Ce(CO_3)_2^- + \frac{3}{2}H_2O + \frac{1}{4}O_2 = 2CO_3^{2-} + 3H^+ + CeO_2 \text{ [4]}$$

In these equations, we assume that when Ce^{3+} oxidizes to Ce^{4+}, it precipitates from the aqueous system as Ce oxide and only Ce^{3+} is incorporated into carbonates. We assume dissolved O_2 was in equilibrium with atmospheric O_2 in shallow-marine environments. Thus, no matter what Ce complexation equation one uses, the Ce oxidation model can be generalized with the following equation using Henry's Law:

$$\log\left(C_{Ce(CO_3)_2^-}\right) = -logK - \frac{1}{4}log(pO_2) + \log\{X\} - YpH \text{ [5]},$$

$where\ K\ is\ equilibrium\ constant,$ activity of water $= 1$, $C_{Ce(CO_3)_2^-} =$ total dissoved trivalent Ce, $X = sum\ of\ HCO_3^-\ and\ CO_3^{2-}$, and $Y = 1, 2, 3$.

Therefore, assiming all other variables are consistenat in eqn [5], we can derive a relationship between total dissolve Ce concentration ($C_{Ce(CO_3)_2^-}$) and partial pressure of oxygen in the atmosphere (pO_2) and pH.

$$C_{Ce(CO_3)_2^-} \sim \frac{1}{\{pO_2\}^{\frac{1}{4}}} \text{ [6]}$$

$$\log\left(C_{Ce(CO_3)_2^-}\right) \sim -YpH \text{ [7]}$$

Assuming that the Nd and Pr concentration in the water column did not change significantly, then the Ce anomaly mainly reflects changes in $C_{Ce(CO_3)_2^-}$. If we use the common definition of Ce/Ce* = Ce × Nd/Pr[2] (*3*) and assume Ce^{3+}, Pr^{3+}, and Nd^{3+} have similar solubilities in the oceans and the relative partitioning of Ce vs. Pr and Nd into carbonates has remained the same through time, the atmospheric pO_2 and seawater Ce/Ce* are reflected in the Ce/Ce* ratios of shallow-marine carbonates.

Therefore, assuming that pH and K did not vary significantly, we can use the following relationship to quantify changes in pO_2 using Ce/Ce*: $\frac{Ce}{Ce^*} \sim \frac{1}{\{pO_2\}^{\frac{1}{4}}}$ [8].

We can then write the equation as

$$pO_2{}^P = \left(\frac{\left(\frac{Ce}{Ce^*}\right)^M}{\left(\frac{Ce}{Ce^*}\right)^P}\right)^4 \cdot pO_2{}^M \qquad [9],$$

in which superscripts P and M indicate the past and modern parameters, where $pO_2{}^P$ is the partial pressure of atmospheric oxygen in the atmosphere at any time in Earth's history, $pO_2{}^M$ is the partial pressure of atmospheric oxygen in the modern atmosphere. We can then calculate $pO_2{}^P$ at any given time of Earth's history if well-preserved carbonate Ce/Ce* values are available.

Sensitivity test for pH and alkalinity

We have derived the general Ce oxidation model with the following equation:

$$\log(C_{Ce}) = -logK - \frac{1}{4}log(pO_2) + \log\{X\} - YpH \ [5],$$

$where\ C_{Ce}$ = total dissolved trivalent Ce, $X = \ sum\ (HCO_3^-, CO_3^{2-})$, and $Y = 1, 2, 3$.

Therefore, depending on alkalinity (combined bicarbonate and carbonate concentrations), *pH* change could have different degrees of influence. The present-day seawater has a *pH* of ~8.1 (M stands for modern in the equation below). If we assume a lower *pH* in the past (P stands for past in the equation below), we get the following *pH*-dependent relationship using equation [5]:

$$pO_2{}^P = \left(\frac{\left(\frac{Ce}{Ce^*}\right)^M}{\left(\frac{Ce}{Ce^*}\right)^P}\right)^4 \cdot \left(\frac{C^P_{(H^+)}}{C^M_{(H^+)}}\right)^Y \cdot pO_2{}^M \quad [10]$$

where Y = 1, 2 or 3 depending on carbonate vs. bicarbonate concentrations in seawater.

Consequently, if *pH* was lower in the past (*4-6*), this would have caused underestimation in our calculations of pO_2. This is shown by the relationship between *pH* and pO_2 in Supplementary Figure 1a, and Y and pO_2 in Supplementary Figure 1b, which illustrate dependence on *pH* and carbonate complexation. For example, if *pH* was 7.9 in the Paleoproterozoic surface ocean, our best estimate for pO_2 would increase from 0.8% to 1.3% or 3.2% for Y equal to 3 instead of 1. This implies that our best pO_2 estimates are the minimum values if seawater *pH* was indeed lower in the past.

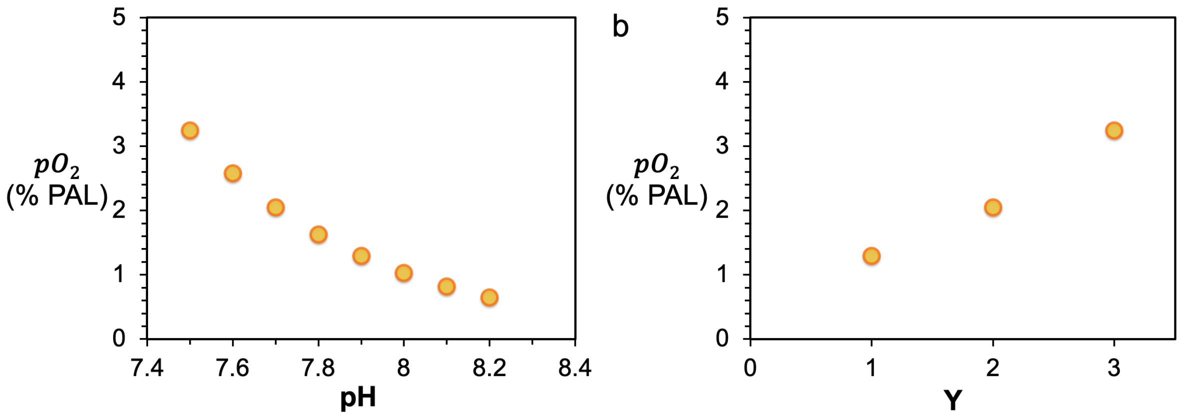

Supplementary Figure 1. a. *pH* vs. pO_2 (% PAL) plot with Y = 1. b. Y vs. pO_2 (% PAL) plot for *pH* = 7.9. Note that the modern seawater pH of 8.1.

Data filtering

Diagenetic alteration can affect the signal recorded by the chemical composition of carbonate rocks; therefore, we carefully screened carbonates for a range of diagenetic effects by combining geologic, petrographic, and elemental and isotopic data. For analyses from the published literature, we compiled data only for samples that are considered to reflect primary depositional environments. For our own analyses, we selected samples with known sedimentological and stratigraphic contexts, most of them were previously analyzed for other proxies. Petrographic analysis was used to select sample areas preserving original sedimentary texture, well-preserved crystalline cements, and the most finely crystalline (e.g. micritic) components, which together suggest minimal recrystallization in the presence of diagenetic fluids; samples were micro-drilled from these spots. To identify the effect of diagenesis not observable in thin sections, we screened samples based on previously intentified classic geochemical tracers of late diagenesis, including major and minor elements (e.g., Ca, Mg, Fe, Mn, and Sr) as well as C and O isotopic signatures. Please see Supplementary Dataset 1 for invididual papers that reported detailed geological, sedimentological, and geochemical information on our samples.

We have observed a correlation between Y/Ho ratios and Ce/Ce* when Y/Ho ratios are less than 20 (Supplementary Figure 2). We recognize that the modern marine carbonates usually have Y/Ho ratios of 44 to 70 (*7*). However, for ancient carbonates, these ratios are common to fall below 44 and even lower than the chondritic value of 28 (*8*). These lower values may come from contamination with non-carbonate phases. Although we adopted the selection criteria used by individual researchers, as published in their original descriptions, we also used an Y/Ho ratio of larger than or equal to 20 as an additional filter for all our own

samples. In addition, we checked REY patterns and Eu anomalies to exclude samples influenced by hydrothermal alteration (*9-11*). For REY patterns, we mostly checked for consistency to exclude samples with non-seawater like patterns. We show two examples – a seawater-like REY pattern and a non seawater-like pattern with Eu anomaly to be excluded in Supplementary Figures 2c and 2d, respectively.

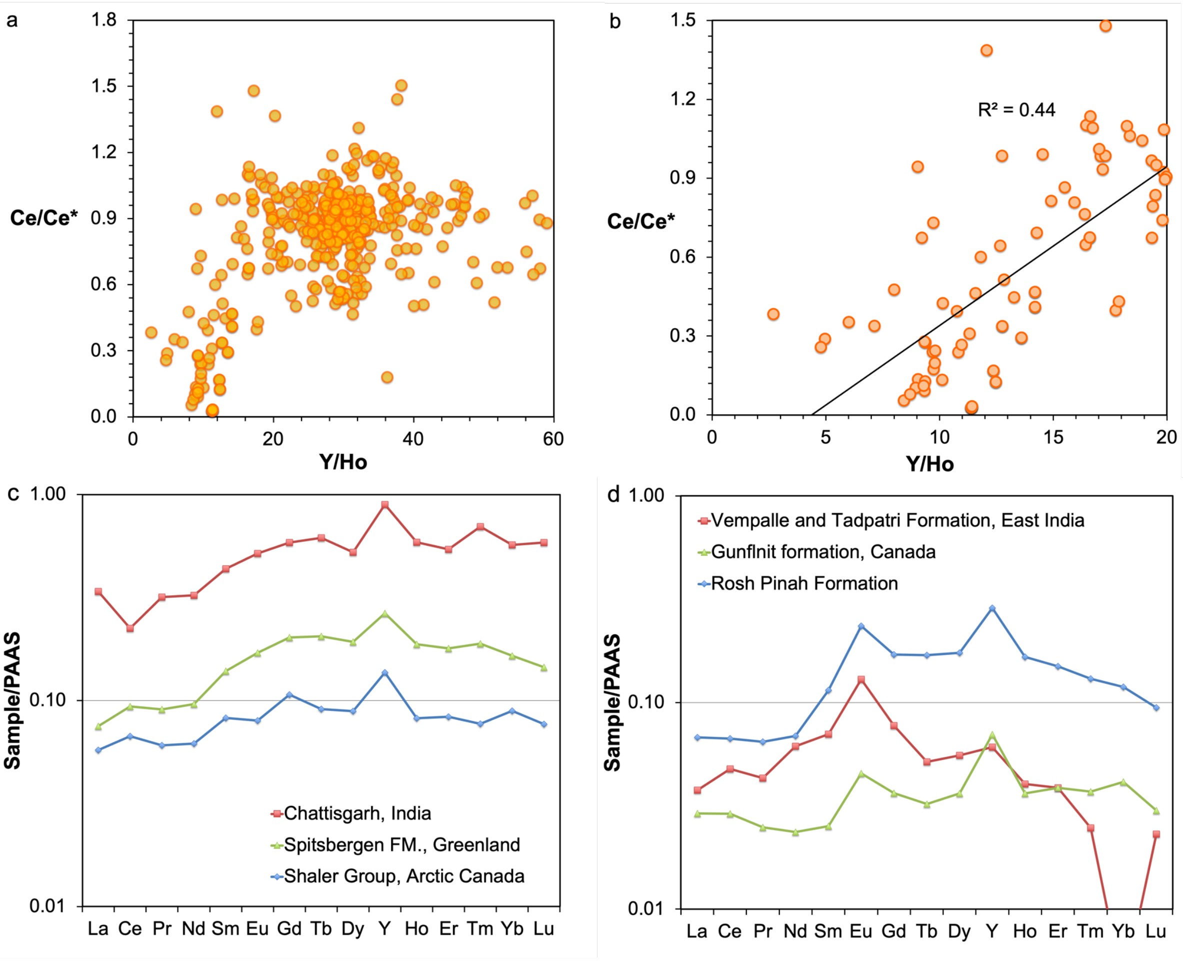

Supplementary Figure 2. a. Ce anomaly (Ce/Ce*) vs. Y/Ho ratio plot for all data in this study. b. In cases with Y/Ho less than 20, Ce/Ce* positively correlates with Y/Ho. c. PAAS (Post Archean Austrialian Shale) normalized REY pattern examples that are included. These patterns are characterized by LREEs depleteion compared to HREEs with high Y/Ho ratios. d. PAAS normalized REY pattern that are filtered out because they display inconsisitent REY patterns without the typical seawater-like feature likely indicating non-carbonate phase contamination and Eu anomalies indicating potential hythrothermal influence.

We have also plotted Ce/Ce* vs. time as well as its temporal distribution combined with grouping based on mineralogy (see Supplementary Figures 3 and 4). We conclude that mineralogy did not significantly influence Ce/Ce*.

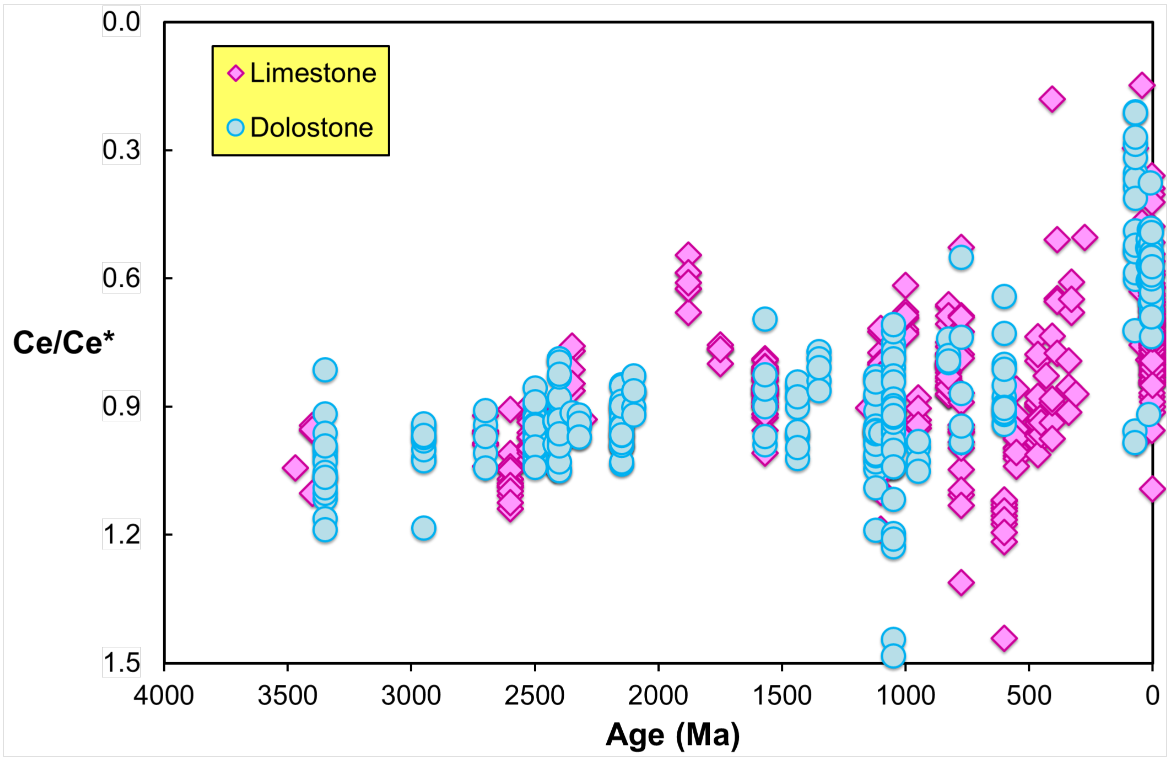

Supplementary Figure 3. Ce anomaly (Ce/Ce*) vs. age plot showing limestone and dolostone separately.

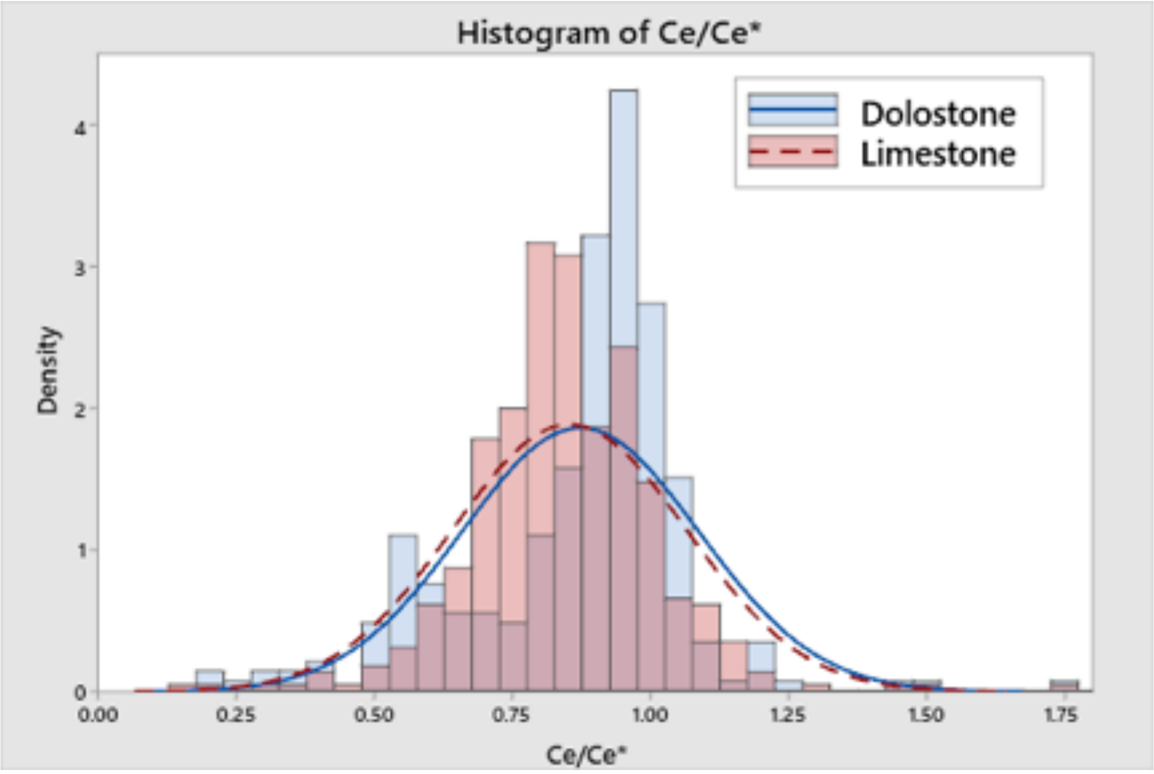

Supplementary Figure 4. Histogram of Ce anomaly (Ce/Ce*) for dolostone and limestone. The mean Ce/Ce* values are 0.87 and 0.85 for dolostone and limestone, respectively. The standard derivation of Ce/Ce* in both groups is 0.21.

Statistical analysis of data

Despite the rigorous application of these screening techniques, we expect that the chemical reactivity of carbonates resulted in at least minimal diagenesis of every carbonate sample we studied. Additionally, since Ce/Ce* shifts towards unity with burial diagenesis *(7)*, and because we are investigating a wide range of samples that represent different conditions of burial, on a variety of time scales, we developed a statistical treatment of our data.

Temporal data sets are subject to biases associated with sampling: recent geological eras are commonly represented by more samples, formations are represented by an unequal number of analyses, and each unit has experienced at least some diagenesis during burial. Furthermore, we do not know exactly how diagenesis, local primary productivity, mineralogy, and kinetics influence Ce/Ce* values. Therefore, we adopted approach to statistically evaluate Ce anomaly data through time, with the goal of investigating the population behavior. First, we divided the entire sample population into five groups of different duration to make sure each group has a statistically significant sample number, where all groups contain n > 100. Groups were also chosen to reflect current models for pO_2 evolution through time (*12, 13*). Besides, we divided into separate groups the early Phanerozoic (Cambrian-Silurian) and late Phanerozoic (Devonian and later) samples to explore existing models concerning atmospheric oxygen increase resulting from the rise of land plants *(14)*. Each group contains samples from at least two different formations. For example, the first bin is from 3.5 to 2.5 Ga, which is the pre-GOE time, where we expect low pO_2, and we observe high Ce/Ce* with little variation. We divide the following samples into 2.5 – 1.6 Ga and 1.6 – 0.65 Ga bins, to have a statistically significant number

of samples in each bin. We then divide the younger group of samples into two bins, a late Neoproterozoic to Early Devonian (650 – 400 Ma) and a mid-Paleozoic to modern bin (400 – 0 Ma) to make self-consistent and statistically meaningful sample subsets.

We then created a box-whisker plot for all data within the five bins (Supplementary Figure 5), where median, 50%, and outliers (outside of three sigmas of the population) of Ce anomaly values were calculated for each bin and are shown with middle lines, blue boxes, and black crosses, respectively, on Supplementary Figure 5. In a second approach, we divided data into the same five bins as in the previous approach, but we plotted histograms for each group (Supplementary Figure 6). Data in nearly all groups follow the normal distribution. Finally, we generated a box-whisker plot for all Ce/Ce* values using 100-million-year bins (Supplementary Figure 7).

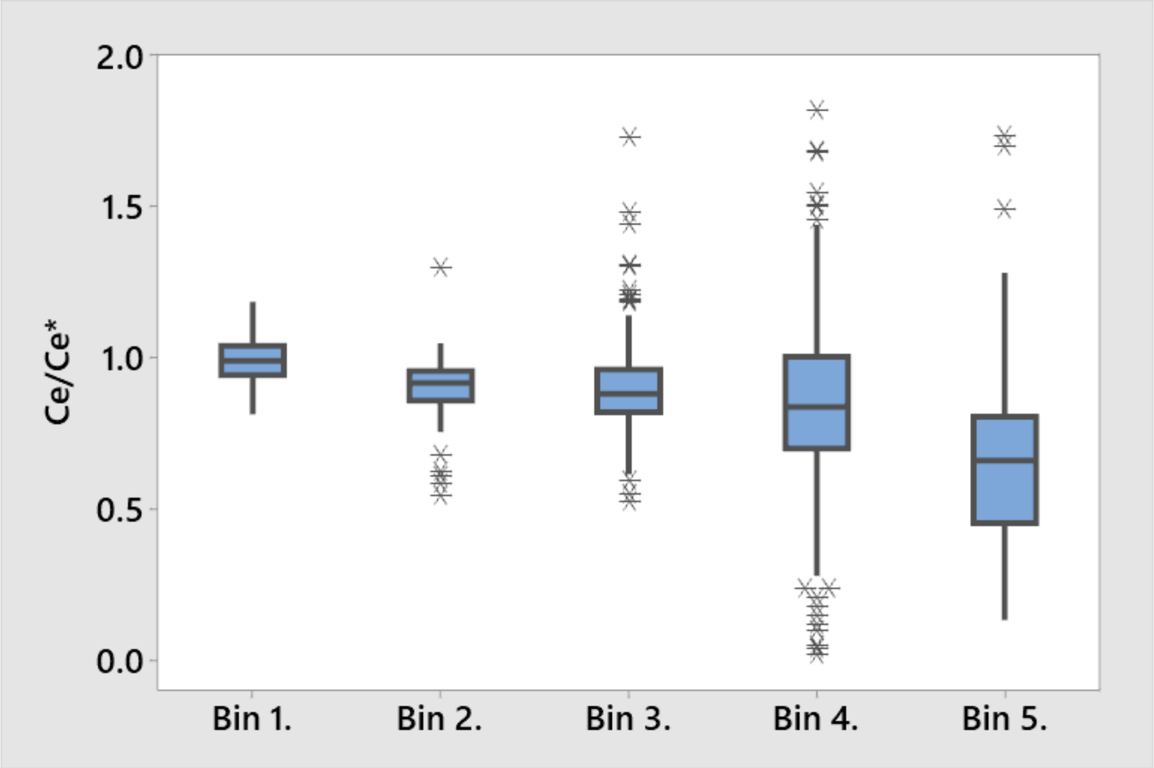

Supplementary Figure 5. Box-whisker plot for all samples. The sample population is divided into five bins (Bin 1. Archean: 3.5 – 2.5 Ga, Bin 2. Paleoproterozoic: 2.5 – 1.6 Ga, Bin 3. Mesoproterozoic – Early Neoproterozoic: 1.6 Ga – 650 Ma, Bin 4. Late Neoproterozoic – Early Paleozoic: 650 – 400 Ma, Bin 5. Mid-Paleozoic to Modern: 400 – 0 Ma). Median values are indicated by the black lines and each individual boxes include 50% samples and whiskers mark the 3 sigma boundaries of the group population. Black crosses fall out of whiskers and are considered outliers.

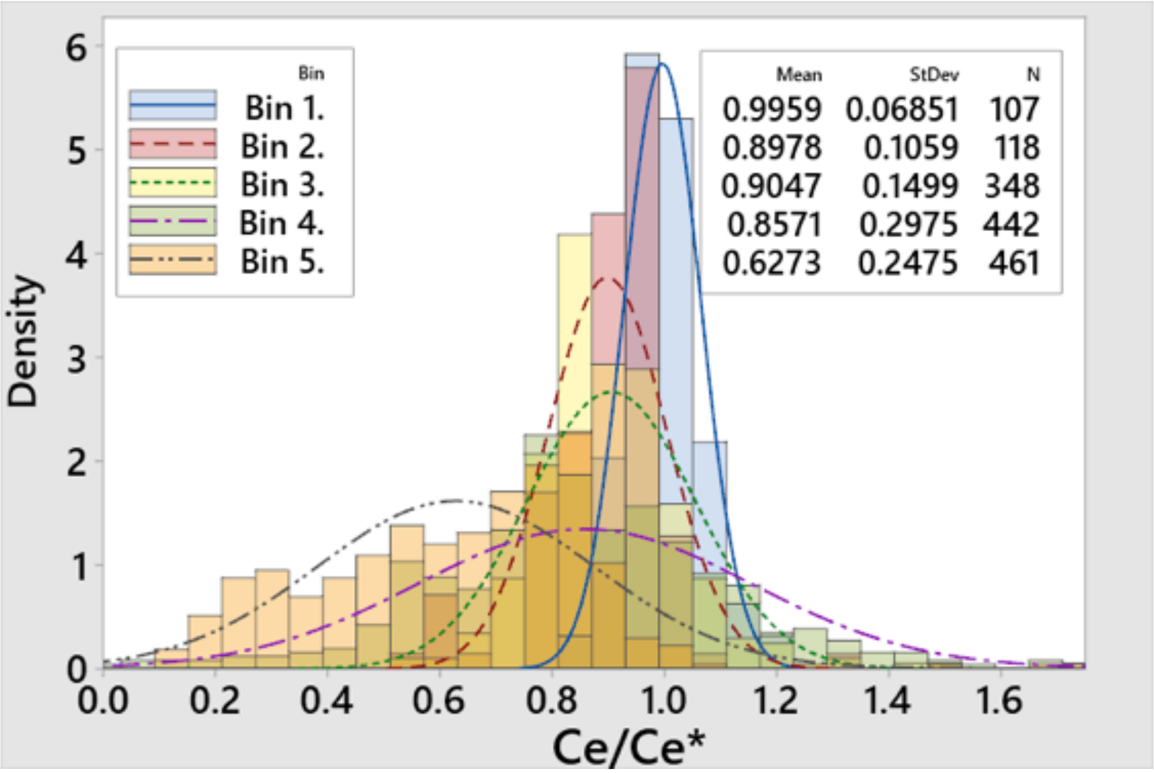

Supplementary Figure 6. Histograms of Ce/Ce* values with normal fitting. We group all data into five age bins (see text above and Supplementary Figure 5 for ages for each bin), and plot distribution and calculate normal fitting for each bin.

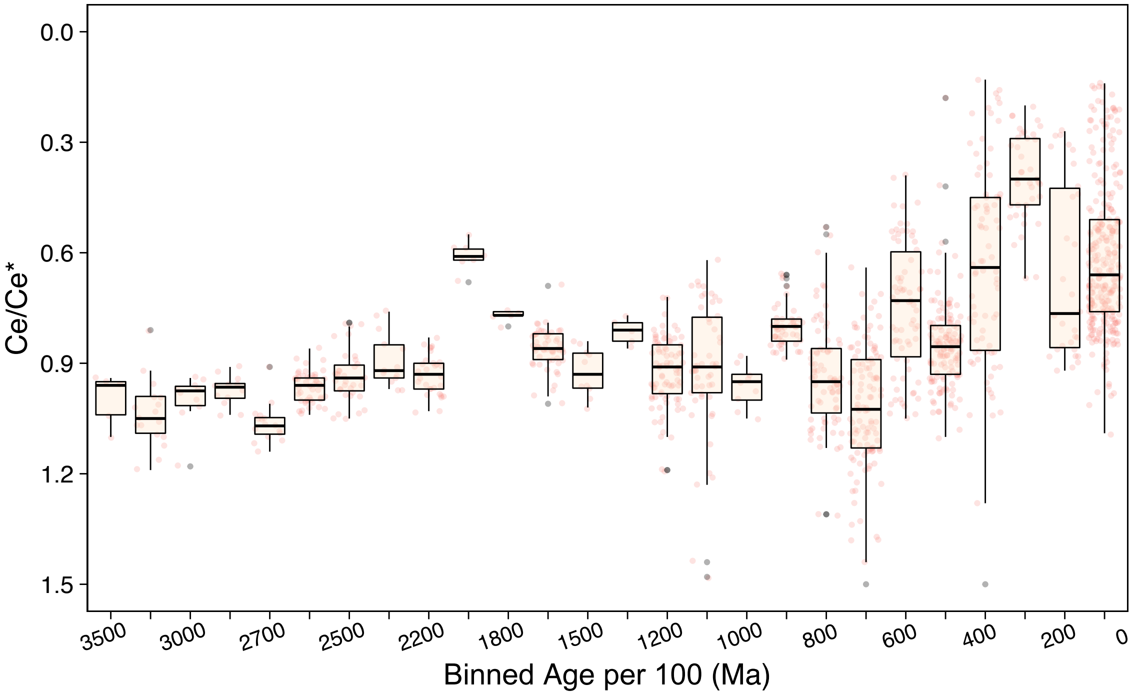

Supplementary Figure 7. Box-whisker plot of Ce/Ce* values for 100 million year bins. Median values are indicated with the middle lines. Each individual box includes 50% of samples and black lines mark the three sigma boundaries of the bin population.

Supplementary References

1. R. H. Byrne, E. R. Sholkovitz, in *Handbook on the Physics and Chemistry of Rare Earths*. (Elsevier, 1996), vol. 23, pp. 497-593.
2. C. Cao, X.-M. Liu, J. D. Owens, H. C. Jenkyns, Late Cretaceous cerium anomalies and globally distinctive redox responses before, during and after Oceanic Anoxic Event 2. *Paleoceanography and Paleoclimatology*, in review (202X).
3. M. G. Lawrence, A. Greig, K. D. Collerson, B. S. Kamber, Rare Earth Element and Yttrium Variability in South East Queensland Waterways. *Aquatic Geochemistry* **12**, 39-72 (2006).
4. J. P. Grotzinger, J. F. Kasting, New Constraints on Precambrian Ocean Composition. *The Journal of Geology* **101**, 235-243 (1993).
5. I. Halevy, A. Bachan, The geologic history of seawater pH. *Science* **355**, 1069 (2017).
6. J. Krissansen-Totton, G. N. Arney, D. C. Catling, Constraining the climate and ocean pH of the early Earth with a geological carbon cycle model. *Proceedings of the National Academy of Sciences* **115**, 4105 (2018).
7. X. M. Liu, D. S. Hardisty, T. W. Lyons, P. K. Swart, Evaluating the fidelity of the cerium paleoredox tracer during variable carbonate diagenesis on the Great Bahamas Bank. *Geochim. Cosmochim. Acta* **248**, 25-42 (2019).
8. M. Bau, Controls on the fractionation of isovalent trace elements in magmatic and aqueous systems: evidence from Y/Ho, Zr/Hf, and lanthanide tetrad effect. *Contributions to Mineralogy and Petrology* **123**, 323-333 (1996).
9. P. Fralick *et al.*, Geochemistry of Paleoproterozoic Gunflint Formation carbonate: Implications for hydrosphere-atmosphere evolution. *Precambrian Research* **290**, 126-146 (2017).
10. H. E. Frimmel, Trace element distribution in Neoproterozoic carbonates as palaeoenvironmental indicator. *Chem. Geol.* **258**, 338-353 (2009).
11. A. C. Khelen *et al.*, Geochemical and stable isotope signatures of Proterozoic stromatolitic carbonates from the Vempalle and Tadpatri Formations, Cuddapah Supergroup, India: Implications on paleoenvironment and depositional conditions. *Precambrian Research* **298**, 365-384 (2017).
12. T. W. Lyons, C. T. Reinhard, N. J. Planavsky, The rise of oxygen in Earth's early ocean and atmosphere. *Nature* **506**, 307-315 (2014).
13. X.-M. Liu *et al.*, Tracing Earthís O_2 evolution using Zn/Fe ratios in marine carbonates. *Geochemical Perspectives Letters* **2**, 24-34 (2016).
14. M. W. Wallace *et al.*, Oxygenation history of the Neoproterozoic to early Phanerozoic and the rise of land plants. *Earth and Planetary Science Letters* **466**, 12-19 (2017).
